# Intraoperative antepulsion of a posterior lumbar interbody fusion cage: three case reports

**DOI:** 10.11604/pamj.2015.20.342.5750

**Published:** 2015-04-10

**Authors:** Davut Ceylan, Can Yaldiz, Kiyasettin Asil, Tibet Kaçira, Necati Tatarli, Aytaç Can

**Affiliations:** 1 Sakarya University School of Medicine, Department of Neurosurgery, Sakarya, Turkey; 2 Sakarya University School of Medicine, Department of Radiology, Sakarya, Turkey; 3 Education and Research Hospital, Department of Neurosurgery, Istanbul, Turkey

**Keywords:** Posterior lumbar interbody fusion cage, antepulsion, interbody cage dislocation, migration of fusion cage

## Abstract

Spinal fusion surgery techniques develop together with technologic advancements. New complications are seen as the result of new techniques and these may be very severe due to spinal cord and vascular structures in the lumbar region. The posterior lumbar interbody fusion cage (PLIFC) was shown to enhance spinal fusion and to prevent pseudoarthrosis due to its basic dynamic characteristics. PLIFC migrations are usually observed during the postoperative period, just after the mobilization of the patient and usually toward spinal canal. Migration to the retroperitoneal region is a extremely rare condition in the literature. In this article we discussed three cases of PLIFC antepulsion into the retroperitoneal region during the intraoperative period.

## Introduction

Spinal fusion surgery techniques develop together with technologic advancements. New complications are seen as the result of new techniques and these may be very severe due to spinal cord and vascular structures in the lumbar region. Consequently, related mortality and morbidity may increase [1, 2]. The posterior lumbar interbody fusion cage (PLIFC) was shown to enhance spinal fusion and to prevent pseudoarthrosis due to its basic dynamic characteristics [3]. In addition, the PLIFC enables the restoration of disc height and decompression of neural structures by providing anterior support to the vertebral column in degenerative lumbar spinal diseases [4, 5].

## Methods

In this paper, we discuss the one-year outcomes of PLIFCs that showed antepulsion into the abdominal cavity during the intraoperative period.

## Results

**Figure 1:**
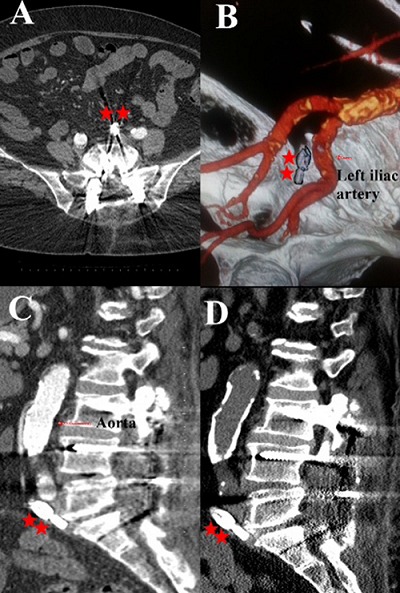
Axial (A) and sagittal (C) images of PLIFC(**) with antepulsion from the L5-S1 intervertebral disc space replaced nearby to the left iliac artery (B) is realized on the control CT performed postoperative first day. No movement change of the cage is seen in the postoperative 1 year control CT (D)

**Case 1:** A 60-year-old female patient was admitted to our clinic with complaints of low back pain and leg pain that increased with walking, as well as neurogenic claudication. She underwent an operation for spinal stenosis and degenerative disc disease between L3-S1. A bilateral transpedicular screw system (BTSS) was placed at the L3-L4-L5-S1 levels. Total laminectomy and bilateral foraminotomy were performed on the L3-L4-L5 spine. A PLIFC was placed without problems through fluoroscopy control after the dura and roots were eliminated and L5-S1 discectomy had been performed; fluoroscopy showed the cage to be located in retroperitoneal region as anterior resistance was not felt during placement of the left PLIFC. The CT images obtained in the early postoperative period confirmed that the cage was in the retroperitoneal region at L5-S1 level (Figure 1).

**Case 2:** A 55-year-old female patient was admitted to our clinic with complaints of neurologic claudication that negatively affected her daily life, as well as numbness and tingling in her both legs. The patient had spinal stenosis and degenerative disc disease between L3-S1 levels and underwent an operation. A BTSS was placed at L3-L4-L5-S1 levels. L4-L5 total laminectomy, superior facetectomy, and bilateral foraminotomy were performed. The right PLIFC was placed without problems through fluoroscopy control after L5-S1 discectomy had been performed by eliminating dura and roots; fluoroscopy control was performed as the cage easily moved to the depth without resistance during the left PLIF cage insertion. The cage was observed to be in the retroperitoneal region at the L5-S1 level. A lumbar CT obtained in the early postoperative period revealed that PLIFC was at the L5-S1 disc space in the left retroperitoneal region (Figure 1).

**Figure 2:**
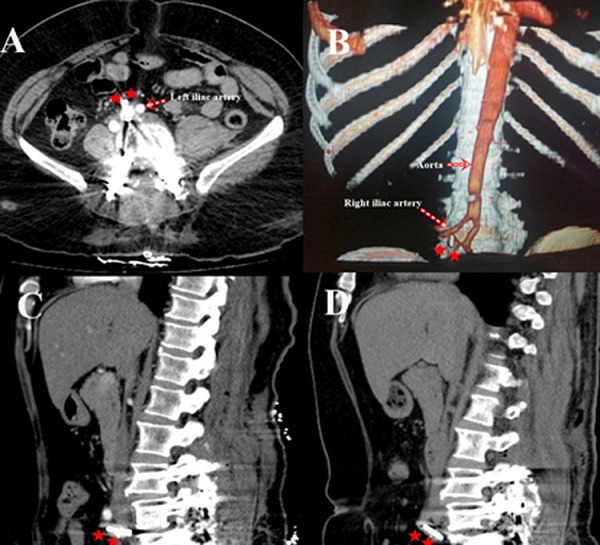
Axial (A) and sagittal (C) images of PLIFC(**) with antepulsion from the L5-S1 intervertebral disc space replaced nearby to the left iliac artery (B) is realized on the control CT performed postoperative first day. No movement change of the cage is seen in the postoperative 1 year control CT (D)

**Case 3:** A 53-year-old female patient was admitted to our clinic with complaints of neurogenic claudication and pain that increased with walking in the lower extremities and decreased when leaning forward. She underwent an operation due to spinal stenosis and degenerative disc disease between L3-S1. A BTSS was placed at L3-L4-L5-S1 levels. L3-L4-L5 total laminectomy, superior facetectomy, and bilateral foraminotomy were performed. Fluoroscopy control was performed as the cage easily progressed to the correct depth without resistance during the right PLIFC placement after L4-L5 discectomy had been performed by eliminating dura and the roots. PLIFC was observed to be located in the retroperitoneal region. The operation was terminated without a cage on the left. PLIFC was observed to be in the right retroperitoneal region at the L4-L5 level on lumbar CT obtained in the early postoperative period (Figure 2).

**Postoperative follow up of the patients:** The patients were closely followed up hemodynamically by the anesthesia team in the perioperative period and general surgery and cardiovascular surgery clinics were consulted for assessment of intra-abdominal organ and/or vascular injuries. Abdominal USG, lumbar spinal CT, abdominopelvic CT, and abdominopelvic CT angiography were performed for elimination of vascular and internal organ injuries in the early postoperative period. No pathologies were detected as the result of these tests (Figure 1 (B), Figure 2, (B)). The hemogram, arterial blood pressure, and pulse were monitored closely in the intensive care unit during the postoperative 24 hours. The patients were mobilized with lumbosacral corsets, and invited for control follow-up at months 1, 3, 6, 9. No injuries were detected in the internal organs and vascular structures. No movement was observed on control CT in the cages that migrated to anterior position in case1 and case 2, but in case 3 minimal movement change with a cranial angulation is seen on the control CT (Figure 1 (D), Figure 2, (D)).

## Discussion

Very severe complications may be seen in spinal surgery due to the proximity of the spinal cord and vascular structures to the operative field. The use of posterior transpedicular screw systems (PTSS) and PLIFC has gradually increased for treatment of spinal instability and/or spinal stenosis and related complications have also increased [1, 2, 5–7]. Classical complications of PLIFC include spinal root injury, dura injury, epidural fibrosis and/or arachnoiditis, infection, and cage migration. PLIFC migrations are usually observed during the postoperative period, just after the mobilization of the patient and usually toward spinal canal [1]. The main cause of posterior migration of PLIFCs is total facetectomy and insufficiency of posterior stabilization [4, 5]. Migration to the retroperitoneal region is a rare condition in the literature. While Ignacio et al. [1] claimed that late abdominal exploration could lead to more severe outcomes in case of antepulsion of PLIFCs, they recommend early exploration and removal of the cage and repair of organ and vascular injuries, if present. We closely monitored hemodynamics in the early postoperative period and verified the absence of intra-abdominal and great vessel injuries through abdominal USG, abdominopelvic CT, and abdominopelvic CT angiography performed in the early period. No complications were observed in the early and late follow-ups. PLIFCs were not removed, thereby preventing complications. Passage of the cage to the retroperitoneal region was in a relatively safer field surrounded by the anterior surface of the spine and outer membrane of the peritoneum. In addition, limitation of low back movements due to long segment posterior stabilization could have contributed to the displacement of the cage.

Lumbar disc prosthesis (LDP) cases dislocated to anterior have been reported in literature and some of these cases were dislocated in the early period and some in the late period. In all these cases, removal is recommended in the early period prior to adhesion development in the operative field. Disc prosthesis developing in the late period may lead to injuries of the small intestine, colon, and ureter, in addition to the complications related to great vessel compression or rupture [8]. Surgical removal is recommended in these cases although it is difficult and may lead to complications [8–10]. The size of the lumbar disc prosthesis is larger than the PLIFC and its borders are sharper, which means it is more prone to future complications even though asymptomatic. Tight retroperitoneal adhesions may increase the risk of vascular and internal organ injury, together with dislocation of the prosthesis as LDP is performed with a retroperitoneal approach, while the PLIFC is smaller and round. Complication risks may be less as it migrates into the retroperitoneal region without adhesions. Ignacio et al. [1] reported that the impaction of the cage by means of a bone packing instrument helped the anterior displacement of the implant to the abdominal cavity. We consider that uncontrolled hammering of the cage or applying excessive power when tightening the screw that provides expansion could lead to antepulsion. A wide range exists for possible anatomic variants of vessels [11, 12]. Common iliac vessels originate at this level in anterior of the L4-L5 [13]. Gstöttner et al. [12] found aortic bifurcation at the level of the L5 vertebral body at a ratio of 61%. The iliac vein confluence occurred mainly at the L5 vertebral body (67%). The left common iliac artery is the most commonly injured vessel in lumbar disc hernia due to its proximity with the L4-L5 intervertebral disc and it proceeds in a medial direction [13]. One of our cases antepulsed at the L4-L5 space and it was on the right. Less vascular injury occurs at L5-S1 as it is directed toward the lateral of both corpus body in lumbar disc surgery [13]. Two of our cases had antepulsed from the L5-S1 space and this was on the left. Parasympathetic nerves originate from the second and fourth segments of the sacral spinal cord (S2-S4). The parasympathetic system is confluent with the sympathetic nerves coming from the hypogastric plexus in the pelvic plexus. Injury of this plexus results in impotence in males. However, our three patients were female and plexus injury was not considered.

## Conclusion

Excessive curettage could lead to tearing of the anterior longitudinal ligament (ALL) and annulus fibrosus when applying PLIFC. The cage should be hammered carefully under scope control. Excessive power should be avoided during tightening of the screw. The L5-S1 region should be closely monitored for acute injury of organs and vascular structures in case of anterior migration of PLIFC although it is a less risky region for injury. We consider that early retroperitoneal explorative surgery is not required unless intra-abdominal organ and vascular injury develop.
